# Use of UHPH to Obtain Juices With Better Nutritional Quality and Healthier Wines With Low Levels of SO_2_

**DOI:** 10.3389/fnut.2020.598286

**Published:** 2020-12-04

**Authors:** Antonio Morata, Buenaventura Guamis

**Affiliations:** ^1^enotecUPM, Universidad Politécnica de Madrid, Madrid, Spain; ^2^Universidad Autónoma de Barcelona, Barcelona, Spain

**Keywords:** emerging technologies, grape must, winemaking, oxidative enzymes, colloidal stability, additives, sulphites

## Abstract

Ultra-high pressure homogenization (UHPH) is a high pressure technique in which a fluid is pressurized by pumping at higher than 200 MPa and instantaneously depressurized at atmospheric pressure across a special valve. The full process takes <0.2 s and the in-valve time is <0.02 s. In the valve, extremely intense impacts and shear forces produce the nanofragmentation of biological tissue at a range of 100–300 nm. The antimicrobial effect is highly effective, reaching easily inactivation levels higher than 6-log cycles even at low in-valve temperatures. At in-valve temperatures of 140–150°C (0.02 s) the destruction of thermoresistant spores is possible. Even when the temperature in-valve can be elevated (70–150°C), it can be considered a gentle technology because of the tremendously short processing time. It is easy to get outlet temperatures after valve of 20–25°C by the expansion and assisted by heat exchangers. Thermal markers as hydroxymethylfurfural (HMF) are not formed, nor are deleterious effects observed in sensitive compounds as terpenes or anthocyanins, probably because of the low effect in covalent bonds of small molecules of the high-pressure techniques compared with thermal technologies. Additionally, intense inactivation of oxidative enzymes is observed, therefore protecting the sensory and nutritional quality of fruit juices and avoiding or reducing the use of antioxidants as sulphites. UHPH can be consider a powerful and highly effective continuous and sterilizing technology without thermal repercussions, able to keep fresh juices with most of their initial sensory and nutritional quality and allowing high-quality and natural fermented derivatives as wine.

## Introduction

**Figure 1:**
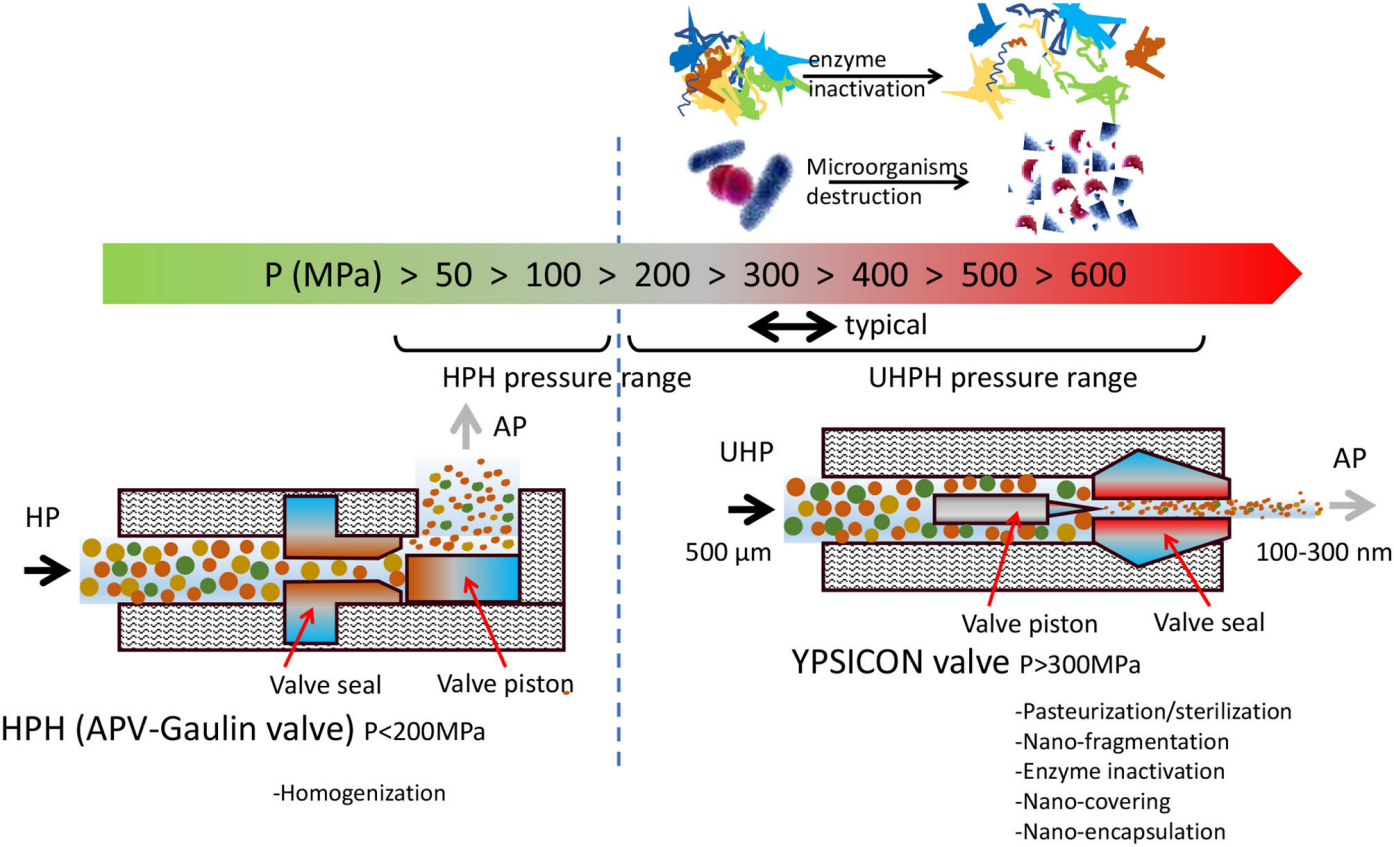
Range of pressures in conventional homogenization vs. UHPH. Effect on enzymes and microorganisms and nano-fragmentation in the UHPH valve.

Currently there are available at commercial and industrial levels two groups of high pressure technologies: (i) discontinuous high hydrostatic pressure (HHP) and (ii) continuous high pressure homogenization including high pressure homogenization (HPH), microfluidization (MF), and ultrahigh pressure homogenization (UHPH). The first group (i) uses a fluid, usually water, as pressurizing media, and the antimicrobial effect is produced by the damage produced in membranes and cell walls by the intense pressures ranging from 400 to 600 MPa, during 3–10 min (1, 2). It is possible to process solid and liquid foods. This technology is discontinuous on batch-mode and unable to inactivate spores (3) and with low effect in enzymes (4, 5). However, it is highly protective with molecules with nutritional or sensory impact, e.g., vitamins, aroma compounds, and pigments. The second group (ii) are continuous technologies in which the liquid is pumped at high pressure. In UHPH, which is the most effective, pressure ranges from 200 to 600 MPa (Figure 1). The antimicrobial effect is produced by the intense impact and shear forces produced in the valve (2, 6, 7). Liquid suffers strong acceleration when pumped at 300 MPa, reaching Mach 2 speed and extremely intense deceleration to almost zero when crossing the valve during <0.02 s. The effect is a full nanofragmentation to a submicro size range of 100–300 nm of whatever biological structure: cells, tissues, and biomolecules including enzymes (8). Depending on in-valve temperature (140°C), even spores are destroyed. UHPH sterilization is an alternative technique to UHT but with lower sensory and nutritional impact.

The objective of this review is to explain the main features of the processing by UHPH and its advantages compared with the conventional hydrostatic pressurization (HHP). Additionally, it described the impact of UHPH in sensory and nutritional quality and the reduction of conflictive additives as sulphites.

## Performance of Continuous UHPH vs. HHP

UHPH is a powerful technique that can be applied in continuous mode to whatever fluid with a size particle lower than 0.5 mm and a viscosity below 2,000 centipoises (9), and therefore can be used in most food fluids. Currently UHPH technologies can be found at the commercial level; one of the most evolved is patented by UAB and exploded by Ypsicon Advances Technologies (10). Liquids are pumped usually at 300 MPa, which can be considered the limit between HPH and UHPH (11), and the antimicrobial effect and enzyme destruction is produced in a special valve (7, 12) produced with special strong materials as tungsten carbide (weaker) or artificial diamond (highly resistant). The homogenizing valve has a few μm in width favoring mechanical interactions with microbial cells (11, 13). In-valve the intense impact and shear efforts produce several effects at nano-scale: nano-fragmentation, nano-covering, nano-encapsulation, and nano-emulsion that can be modulated to produce sterile or pasteurized foods (2, 7, 8, 14, 15), to increase the accessibility of nutrients and health-promoting compounds (16) and to develop innovative foods with improved colloidal structure or novel properties.

Pressure range in UHPH is 200–600 MPa; however, at 300 MPa the microbial and enzyme inactivation is more intense. Pressure higher than 300 MPa does not produce more intense effects but sometimes increases the level of wearing in-valve and other components. Concerning the temperature, most of the applications can be done at pasteurization or lower temperatures (<70°C). This includes enzyme inactivation, microbial destruction, nano-emulsion, and nano-covering (7, 8, 14, 17).

Concerning microbial inactivation at low pasteurization temperatures (<70°C) or even at room temperature, but considering the shorter processing times of UHPH (0.02 s in-valve and around 0.2 s of total process), it is possible to eliminate vegetative cells of fungi, yeast, and bacteria at populations of 6-log CFU/mL or higher (8, 14). The short in-valve time at this temperature guarantees the absence of thermal markers as hydroymethylfurfural (HMF) (8).

The elimination of bacterial endospores requires higher temperature 140–150°C (7, 18); however, thermal effect is very soft considering the short processing time that is lower than 0.2 s for the full process (14). This allows a higher sensory quality than traditional UHT.

The main features of UHPH in comparison with HHP concern the processing mode, effects on food components, antimicrobial capacity, impact in nutritional and sensory properties, and evaluation of thermal degradation as shown in Table 1. UHPH shows some advantages concerning gentle impact in food quality with a more effective antimicrobial and anti-enzymatic performance.

**Table 1:** Comparative features of UHPH and HHP.

**Effect**	**UHPH**	**HHP**	**References**
Operating mode	Continuous pumping. Treatment time 0.2 s	Discontinuous pressurization with water. Treatment time 3–10 min	(2, 7)
Effect on covalent bonds in small molecules with sensory impact	Unaffected	Unaffected	(19)
Molds and yeasts control	Highly effective at 300 MPa	Highly effective at >400 MPa/5–10 min	(1, 8, 14, 20–23)
Bacteria	Highly effective at 300 MPa	Effective at >600 MPa/5–10 min	(1, 14, 20, 22, 23)
Bacterial endospores	Highly effective at 300 MPa, if in-valve Temperature 140°C 0.2 s	Not applicable. Effective at >1,000 MPa/5–10 min	(7, 18, 24)
Effect on biopolymers	Intense fragmentation. 100–300 nm.	Starch gelatinization. Protein denaturation.	(7, 19)
Oxidative enzymes	Strong inactivation at 300 MPa. PPO inactivation >90%. Absence of browning during more than 5 days in air exposed juices	Weak. Variable, usually needs temperature assistance	(8, 14, 25, 26)
Thermal markers (HMF)	Undetected	Lower than in thermal treatments	(8, 27)
Antioxidant capacity	Increased >150%	Non differences-slight reduction	(14, 28, 29)
Vitamins	Preserved	Preserved	(30, 31)
Terpenes and aroma molecules	Unaffected	Unaffected	(8, 32)
Anthocyanins	Unaffected. Increased extraction from skin colloidal particles	Unaffected. Improved extraction from grapes	(1, 14, 25, 28)
Polyphenols	Improved extraction from apple and grapes	Non-significant differences in total phenolics and flavonoids. Improved extraction from grapes.	(1, 18, 33)
Sensory profile	Unaffected. Better fruitiness	Unaffected	(8, 33–35)
New fermentative biotechnologies as use of non-*Saccharomyces* and yeast-bacteria co-inoculations	Better implantation and lesser competitiveness with indigenous microbiota	Better implantation and lesser competitiveness with indigenous microbiota	(1, 14, 23)
Release of yeast assimilable nitrogen (YAN) and nutritional properties of must	Increased extraction from juice cell fragments. Favors the formation of fermentative esters.	Not described	(14)
Protein digestibility	Improved	Improved	(36, 37)
Allergenicity	Decreased	Decreased	(36, 38, 39)
Colloidal and color stability.	Improved. Higher stability of nanofragmented particles. Color stability. Better protein stability and lower haze formation.	Unaffected. Better protein stability and delayed protein haze.	(8, 14, 15, 34, 36, 40, 41)

## Elimination of Spoilage Microorganisms in Must and Wines and Improved Application of New Biotechnologies

Spoilage yeasts that can be present in grapes, such as *Brettanomyces*, are easily destroyed by UHPH processing (14). Lactic or acetic undesired bacteria that can develop in the future wine increasing volatile acidity, or forming biogenic amines or ethyl carbamate, will be eliminated from the grape must using UHPH, ensuring a healthier wine. The use of 300 MPa and temperatures of 60–100°C for 0.2 s eliminate vegetative bacterial cells (8, 14). Sporulated forms need higher temperatures (6, 7, 18). Toxins as patulin were not directly affected by UHPH; however, a significant decrease can be observed in UHPH juices during storage (18).

There is facilitation of new biotechnologies as use of non-*Saccharomyces* yeasts and yeast-bacteria co-inoculations by a better implantation of the starters in absence of indigenous competitive microorganism (14, 23).

## Destruction of Oxidative Enzymes (PPO) and Retention of Antioxidant Capacity in Juices

HPH can be used to modulate enzymatic activity (42), but at higher pressure using UHPH the enzymes can be denatured. UHPH processing is able to disrupt quaternary structure of proteins (43). The intense inactivation of oxidative enzymes such as polyphenol oxidase (PPO), usually higher than 90% (14, 18), produces wines with antioxidant capacities increased more than 150% regarding controls (8, 14). Preserved antioxidant capacity has also been observed in apple or strawberry juices (30, 44). Moreover, the stability of vitamin C in HPH and UHPH treatments has been confirmed (30, 45). Additionally, UHPH grape must can be exposed to air with high exchange surfaces (>1 cm^2^/mL) without experimenting oxidations or browning processes during several days. The inactivation of pectinmethylesterase has been also observed in apple juices as what contributes to the colloidal stability of turbid juices (18).

The impact of UHPH in the wine industry is the potential reduction of sulfur dioxide (SO_2_) in wines, opening the possibility to produce wines with 0 mg/L of sulphites by inactivating oxidative enzymes but also destroying spoilage microorganisms (8, 14). For juices both the antimicrobial and antienzyme effect facilitate the production of low processed juices that can keep their sensory profile in absence of antimicrobials and antioxidative chemical products for long periods from months to years. The production of SO_2_ free red wine was studied using discontinuous processing by HHP obtaining a good sensory quality (46). However, recently, it has been reported that it is necessary to have at least 60 mg/L of SO_2_ to preserve quality in red wines processed at 350 MPa during 10 min at 8°C (47). Wines with less of 60 mg/L were found both less aromatic and with lower contents of anthocyanins.

## Sensory and Nutritional Quality

Concerning sensory and nutritional quality, the short thermal effect produced by the in-valve temperature is not affecting the degradation of aroma compound as terpenes (8) or delicate pigments as anthocyanins (25, 48). Vitamin contents remain unaffected. Vitamin C contents remain unaffected after UHPH processing in apple juices (18). Thermal markers as HMF are not detected after the UHPH processing (8, 14).

It has been observed that delicate aroma compounds as several terpenes from a Muscat juice are not affected by UHPH treatments at 300 MPa, 65°C, processing time lower than 0.2 s. Concentrations of linalool, terpinen-4-ol, epoxylinalool, β-citronellol, geraniol, α-terpineol, cis-linalool oxide did not show significant differences with the unprocessed controls (8). Also, differences were not found in the pool of polyoxygenated terpenes. Additionally, the sensory panel also did not detect significant differences in the aromatic varietal profile.

Extraction of phenolic compounds can be improved significantly when juices with pulps or skin fragments are processed by UHPH. The intense nanofragmentation of colloids that are reduced to a size range of 100–300 nm increase the extraction of flavonoids and phenols from solids (18).

The color of white musts is improved by the intense inactivation of oxidative enzymes, making it possible to produce clear and pale juices from white grape varieties (8). Furthermore, the color remains pale even after several days of intense exposure to air.

The intense nano-fragmentation on grape cell walls produces a release of nitrogen compounds that increase the yeast assimilable nitrogen (YAN) available with positive effects in yeast nutrition during fermentation but also affecting the formation of fermentative fruity and floral esters (14).

In vegetal beverages of soya and almond processed by UHPH, there have been observed a better protein digestibility and a lower allergenicity (36). The effect is probably due to denaturation of the protein structure. Contents of lysine were stable after the treatment.

## Highlight of Future Directions

The inactivation of spores at lower temperatures can be optimized probably by a new design of special valves built with new materials increasing the impact and shear forces. New geometries of valves increase the mechanical effects, promoting fluid jet impact, cavitation, and extreme shear efforts. Emerging materials also can help to improve the possibilities of processing abrasive foods as high fiber or high viscosity juices and smoothies. The conventional tungsten carbide alloy used in the valves must be substituted by ceramic coverings or artificial diamond seats and needles. There must be better knowledge of the nanoscale processes, and how to manage and monitor the nano-covering, nano-encapsulation, and nano-emulsion processes. The co-injection of different fluids at ultrahigh pressure promotes the formation of interactions among proteins, polysaccharides, and lipids creating nano-structures able to content and to increase the bioavailability of nutraceutical compounds. Using UHPH, it is possible to produce functional foods by the nanoencapsulation and stabilization of nutritional or sensory compounds. Frequently, these nano-encapsulates are more stable in solution or colloidal dispersion creating food fluids with same appearance in the long term without coalescence phenomena. Development of high throughput UHPH machines able to process more than 10,000 L/h is another key parameter to reach industrial scale-up. Currently, there are industrial UHPH devices able to process at 10,000 L/h working at 300 MPa, just with a single pump, but it is necessary to have several pumps to increase this processing flow. There must be development of truck portable UHPH devices to rent the processing technology to be used in seasonal food industries. The elimination of chemical preservatives as SO_2_, sorbates, benzoates, and others can help to reach a key objective in the food industry: cleaning labels.

## Author Contributions

AM: conceptualization, writing draft, revision. BG: writing, supervision. All authors contributed to the article and approved the submitted version.

## Conflict of Interest

The authors declare that the research was conducted in the absence of any commercial or financial relationships that could be construed as a potential conflict of interest.
